# TECHNOLOGY AND AGING FUTURES: DISCOVERING THE CONSUMER ELECTRONICS SHOW (CES) 2019

**DOI:** 10.1093/geroni/igz038.081

**Published:** 2019-11-08

**Authors:** Stephen Katz

**Affiliations:** Trent University, Toronto, Ontario, Canada

## Abstract

The growing field of technology and aging or gerontechnology has largely been considered from a health perspective on technological intervention to ameliorate conditions of isolation, disconnection, inactivity, and loneliness, as well as provide efficient alert systems, transportation coordination, and emergency services. Contesting the image of a ‘digital divide’ separating younger from older generations, the recreational industry has also produced a seniors market of technological games, toys, apps, exercises, and social media. The four papers in this symposium, however, are individual critical reflections by a group of social scientists who visited the Consumer Electronics Show (CES) in January 2019 (Las Vegas) as part of an ethnographic project about the politics of the technical turn in gerontological studies. In particular, the authors gathered evidence from the CES to support their interests in four trends: a) The collecting, aggregating, and sharing of personal data by home surveillance, artificial intelligence monitoring, and self-tracking systems for commercial, insurance and work-place purposes, b) The popularization of healthy lifestyles based on technical and exclusionary models of ‘smart’, ‘fit’, and ‘optimal’ standards, c) The technical rhetoric that infuses designs for efficiency, speed, and convenience with anti-aging and ageist ideologies, d) The challenges to older people to manage their lives against the health risks, interventions, and expectations posed by technology-driven austerity programs. The papers have in common their creative interpretations of CES materials and shared concern about the many older groups whose insufficient access, skill, and resources will deny them participation in the technological imaginary of aging futures.

